# Genome-wide Analysis of Large-scale Longitudinal Outcomes using Penalization —GALLOP algorithm

**DOI:** 10.1038/s41598-018-24578-7

**Published:** 2018-05-01

**Authors:** Karolina Sikorska, Emmanuel Lesaffre, Patrick J. F. Groenen, Fernando Rivadeneira, Paul H. C. Eilers

**Affiliations:** 1 Department of Biometrics, Netherlands Cancer Institute, Amsterdam, The Netherlands; 2 Leuven Biostatistics and Statistical Bioinformatics Centre, Leuven University, Leuven, Belgium; 3 Erasmus School of Economics, Erasmus University, Rotterdam, The Netherlands; 4 Department of Internal Medicine, Erasmus Medical Centre, Rotterdam, The Netherlands; 5 Department of Biostatistics, Erasmus Medical Centre, Rotterdam, The Netherlands

## Abstract

Genome-wide association studies (GWAS) with longitudinal phenotypes provide opportunities to identify genetic variations associated with changes in human traits over time. Mixed models are used to correct for the correlated nature of longitudinal data. GWA studies are notorious for their computational challenges, which are considerable when mixed models for thousands of individuals are fitted to millions of SNPs. We present a new algorithm that speeds up a genome-wide analysis of longitudinal data by several orders of magnitude. It solves the equivalent penalized least squares problem efficiently, computing variances in an initial step. Factorizations and transformations are used to avoid inversion of large matrices. Because the system of equations is bordered, we can re-use components, which can be precomputed for the mixed model without a SNP. Two SNP effects (main and its interaction with time) are obtained. Our method completes the analysis a thousand times faster than the R package **lme4**, providing an almost identical solution for the coefficients and p-values. We provide an R implementation of our algorithm.

## Introduction

Genome-wide association studies with longitudinal phenotypes create opportunities and challenges. On the one hand we can identify genetic variants that are associated with development of traits over time. On the other hand statistical analysis gets more complicated, because (linear) mixed models have to be used.

In this paper we discuss the application of the linear mixed model to repeated measures, collected on unrelated individuals. We assume that the number of measurements per person is just a handful, allowing to model only a linear evolution of the trait over time. In a genome-wide analysis the mixed model has to be fitted for every SNP. It contains fixed effects for time, SNP, and their interaction, and possibly other covariates; it has random intercept and slope for change over time. Of main interest is the time x SNP effect, but multiple observations per individual also increase the power to detect a statistically significant main SNP effect.

A mixed model assumes that some model parameters, in the present case intercept and slope per individual, have been drawn from a (normal) distribution with unknown variance. Also unknown is the variance of the observation error. Once these variances are known, it is straightforward to estimate individual slopes and intercepts. The hard work for mixed models is estimating the variances. Common software, like SAS PROC MIXED and **lme4** in R do this efficiently, using special algorithms. It takes approximately 2.0 seconds to fit a mixed model for several thousand individuals. For a single application this is fast, but for GWAS it is far too slow. Fitting one million mixed models, one for each SNP, would take several weeks of non-stop computation. This assumes that the overhead of accessing the SNP data is negligible, which usually is not the case.

We emphasize that analysis of longitudinal data is different from analysis of cross-sectional outcomes where mixed models are used either to estimate heritability^1,2^ or to correct for hidden correlation due to population stratification^3,4^. Extensive work has been done on how to speed up computations in the latter case, see e.g.^5,6^. Unfortunately it does not solve our problem; see the Discussion.

In an earlier effort, we proposed the conditional two-step (CTS) approach^7^, which summarizes the developmental pattern of a trait as an individual slope, reducing the dimensionality of the data to one pseudo-observation per individual. This allows the use of our fast GWAS algorithm^8^ to obtain an approximate *p*-value for the interaction between SNP and time.

Here, we present a new algorithm for Genome-wide Analysis of Large-scale Longitudinal Outcomes using Penalization (GALLOP) which swiftly computes coefficients and p-values for cross-sectional and longitudinal SNP effects. To arrive at an almost exact solution we exploit several properties of the model. The effect of a SNP generally is (very) small. We estimate the variances in the mixed model without any SNP and assume that they will not change when a SNP is added. This assumption will lead to conservative p-values in case of non-zero SNP-effects. The magnitude of this imprecision is explored in the Results section. Using the equivalence between a mixed model and penalized least squares, a large system of linear equations can be set up. This system is very sparse (it contains many zeros) and only the last rows and columns change from SNP to SNP. With careful organization of the computations a solution is obtained very quickly. No special programming tricks are needed, our program (about 85 lines) is written in pure R and achieves a speed-up by three orders of magnitude, compared to brute-force application of **lme4**. Thanks to the sparseness of the equations, memory use is modest.

Quick access to SNP data is crucial and we also discuss it. An R implementation of GALLOP algorithm is provided. Simulated and real data are used to illustrate performance.

## Results

Two characteristics of our method are of main interest: high speed and accuracy as compared to *lmer* function in the R package **lme4**. We assessed them via a simulation study and using real data.

In the simulation study exploring precision we generated 200 longitudinal data sets on the basis of the mixed model (Equation (3) in the Methods section) using the following settings:

- n = 2000, k = 4, 3 additional covariates
- Measurements occasions (*n* × *k* vector of *t*_*ij*_’s) drawn from a uniform distribution between 0 and 10
- Covariates assumed to be independent, time-varying, and drawn from ${\mathscr{N}}$ (2, 0.5)
- Coefficient for fixed effects: *β*_0_ = −2.6, *β*_1_ = −1.9, *β* ^COV^ independent drawn from ${\mathscr{N}}$(0, 1)
- SNP effects: *β*_2_ and *β*_3_ independent, 200 equally spaced values between 0 and 1
- Variance-covariance matrix of random effects: $$D=(\begin{array}{cc}1 & -0.2\\ -0.2 & 1\end{array})$$ and measurement error *σ* = 2.5
- SNPs drawn from a uniform distribution between 0 and 2

Data sets used to evaluate computation times were generated in a similar manner with sample sizes varying from 1k to 10k with increment of 1k. For each sample size 1000 SNPs were analyzed to summarize computation time.

**Figure 1:**
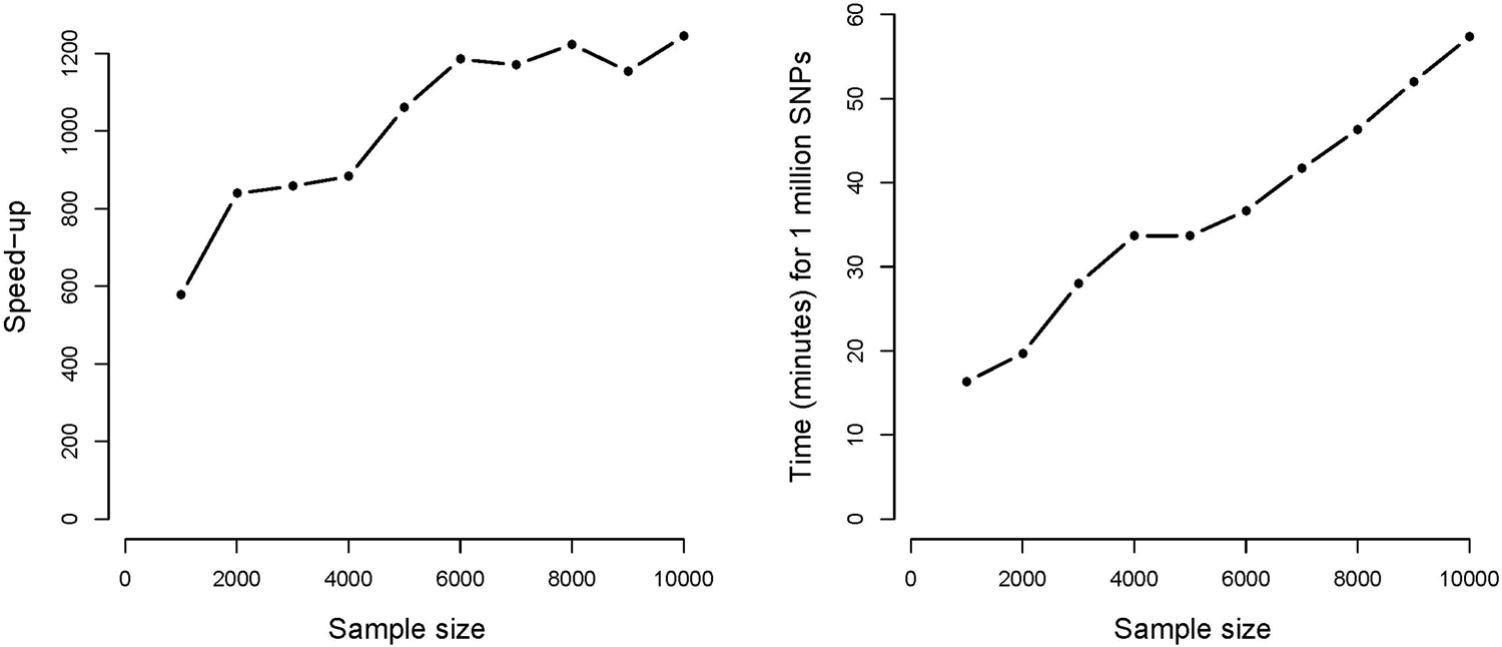
Speed-up compared to the *lmer* function in R. Results based on the simulated data for 1000 SNPs, 4 time points and 3 covariates. Performed on a 64-bit Windows running on a laptop with CPU @ 2.3 GHz and 6 GB RAM.

Results of the simulation study assessing computation times are shown in Fig. 1. The speed-up is linear in the number of individuals. For a genome-wide association study with 5000 individuals our methods finishes the analysis a thousand times faster. A genome-wide scan for 1 million SNPs of a phenotype, collected on 5000 individuals measured on 4 occasions, takes about 30 minutes, instead of 3 weeks when using the package **lme4**. The speed-up depends also on *k*. This is mainly attributed to the fact that **lme4** requires expansion of the SNP vector.

Results of the simulations exploring accuracy are summarized graphically. Based on our theoretical derivations described in the Methods section we know that a non-zero main SNP effect affects the approximation of the variance of the random intercept. Similarly, the size of the interaction influences the variance of the random slope. On the other hand, genome-wide association studies typically show only very small SNP effects which barely contribute to the improvement of the goodness of fit. We ran simulations to explore the practical dangers and consequences of using the approximate variances. Despite the difficulties in defining the variance explained in mixed models we used a simple definition quantifying predictive power as the ratio ${R}^{2}=1-||y-\hat{y}|{|}^{2}/||y-\bar{y}|{|}^{2}$, where $\hat{y}$ stands for the fitted values and $\bar{y}$ for the average of *y*.

**Figure 2:**
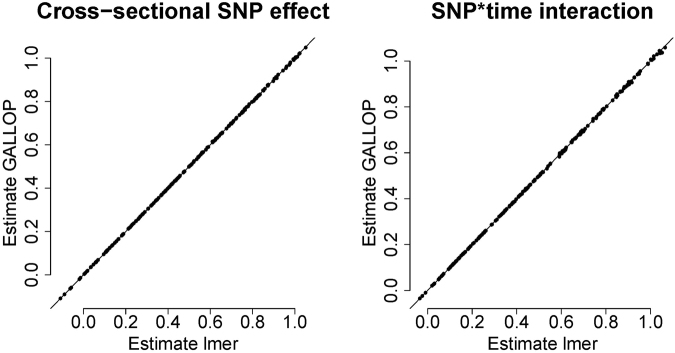
Simulation study. Accuracy of the coefficients computed by GALLOP compared to *lmer*.

**Figure 3:**
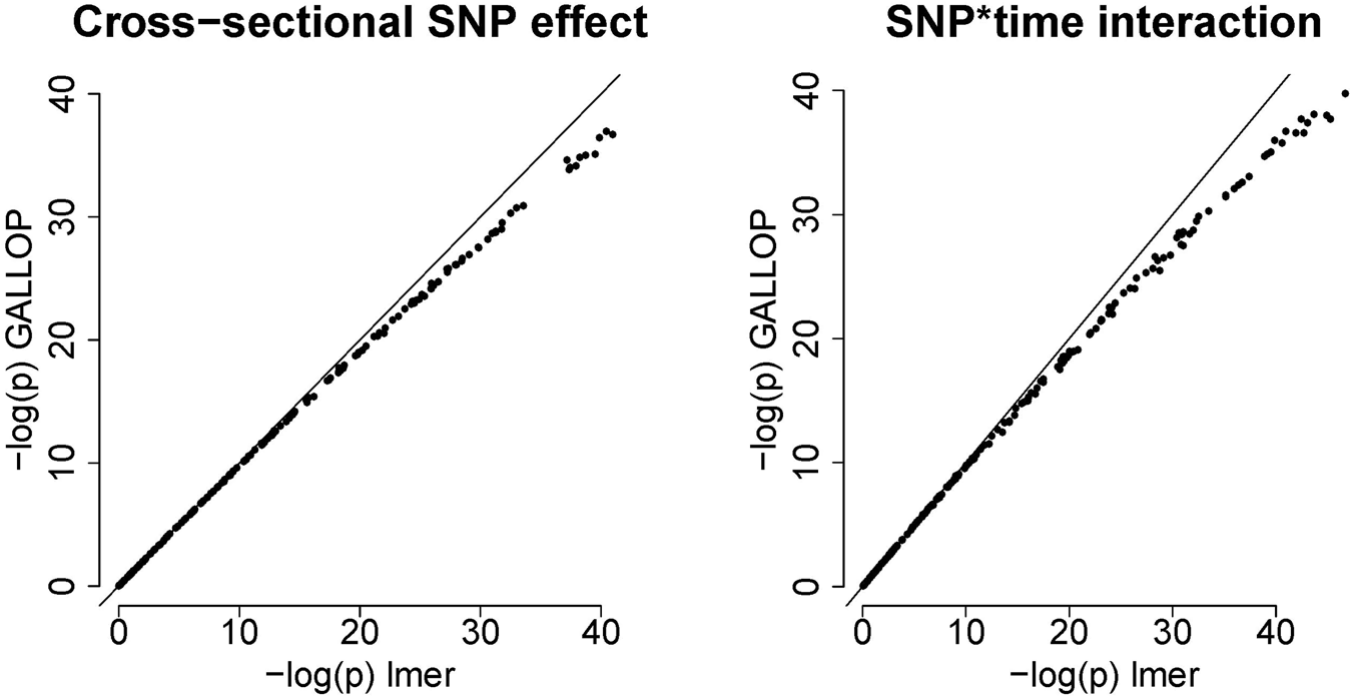
Simulation study. Accuracy of the p-values computed by GALLOP compared to *lmer*.

The estimates are very accurate throughout the entire range of observed values (Fig. 2). The standard errors are somewhat overestimated for the larger values of *β*, which is expected as variances of random effects are inflated due to omitted SNP effects. However, the main interest in GWAS always lies in *p*-values (Fig. 3). These are almost exact (and never too optimistic) in the common GWA-range (0 < −log 10 (*p*) < 7). That eliminates the danger of finding too many false positive results. Due to overestimated standard errors, the −log 10(*p*) for larger betas are too pessimistic. Nevertheless, they increase monotonically with larger effect sizes, just with bias downward with respect to the −log 10 (*p*) from *lmer*. This loss of power can be solved by lowering the threshold for “GWAS significance” and repeating the analysis for promising SNPs with the correct model. In our simulation study, to find all SNPs for which −log 10 (*p*_*lmer*_) > 7.3 we had to use the threshold −log 10 (*p*_*GALLOP*_) > 7.05. In our simulation study the maximum contribution to *R*^2^ of the SNP effects around 6%.

**Figure 4:**
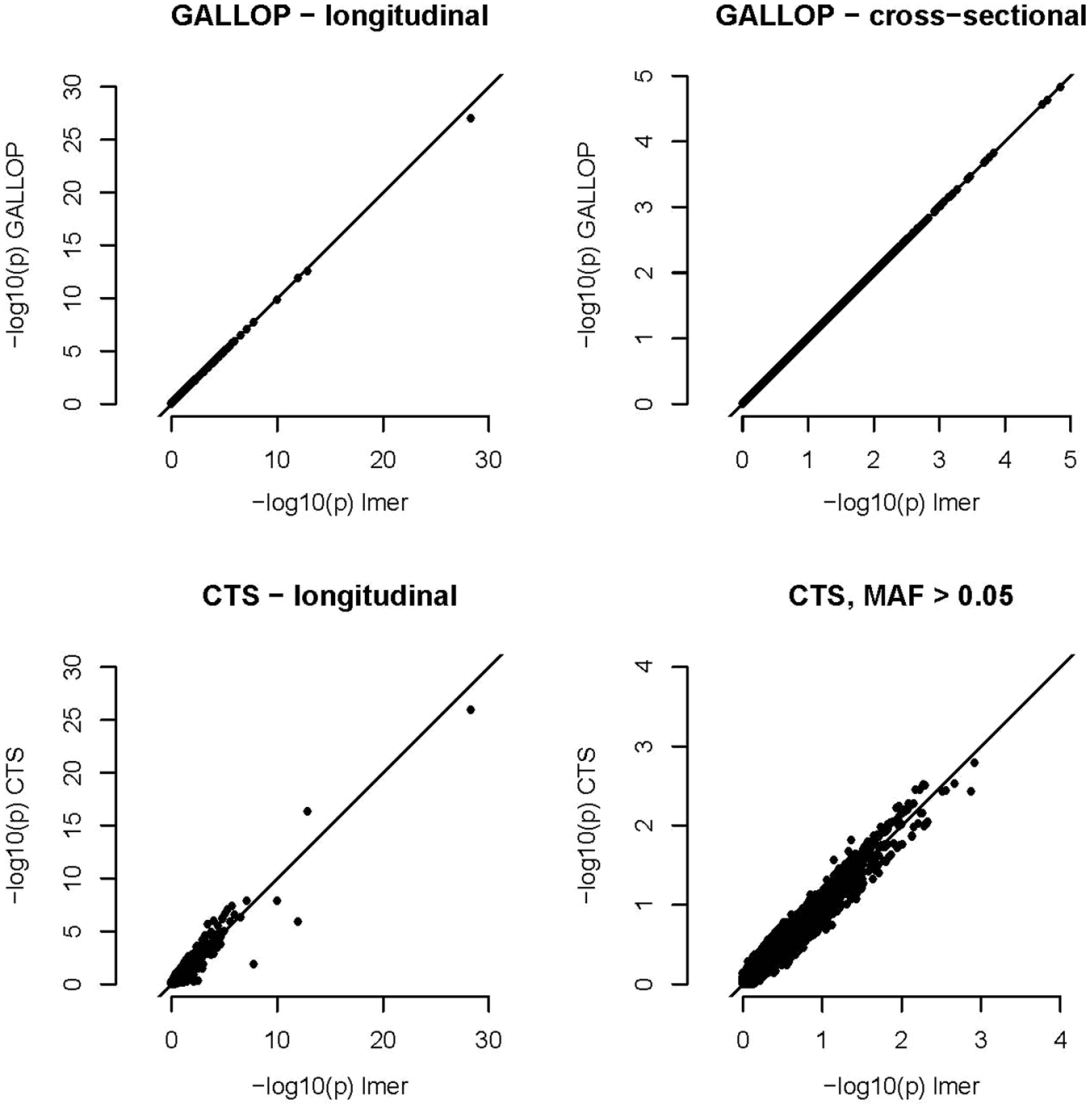
BMD data. Accuracy of the p-values for the GALLOP and CTS.

To confirm the accuracy of GALLOP on real data, we used the BMD data from the Rotterdam Study^9^. Details on the longitudinal BMD data set are provided in ref.^7^. For this analysis we used SNP data imputed according to the 1000 Genomes Project, which were stored per (part of) a chromosome as DatABEL files. To test our algorithm we used one of the files, which contained 97384 SNPs. We performed the association analyses with three methods: GALLOP, CTS, and *lmer* (only for 20 K SNPs). Comparison between p-values is shown in Fig. 4. CTS approach gives a good approximation of the *p*-values for longitudinal SNP effect, which coincide with our previous results on the real and simulated data. However, *p*-values from GALLOP are basically exact for main and longitudinal effect, irrespective of minor allele frequency. The analysis took 3.5 minutes for GALLOP, 40 seconds for CTS and 48 hours (extrapolated time based on the 20 K SNPs) for *lmer*, respectively.

## Discussion

We presented a new algorithm for fast genome-wide analysis with longitudinal data. Our method runs a thousand times faster than **lme4**, which is the fastest option in R. This speed-up is achieved by combining an accurate approximation with a careful implementation. We showed that our method provides practically exact results. In case of doubt one can always do a full mixed model analysis for each of the most significant SNPs. Generally this is a small number; in case of BMD data 6 genotypes for any MAF reached threshold of −log 10 (*p*) > 7; so the extra computation time is negligible.

**Figure 5:**
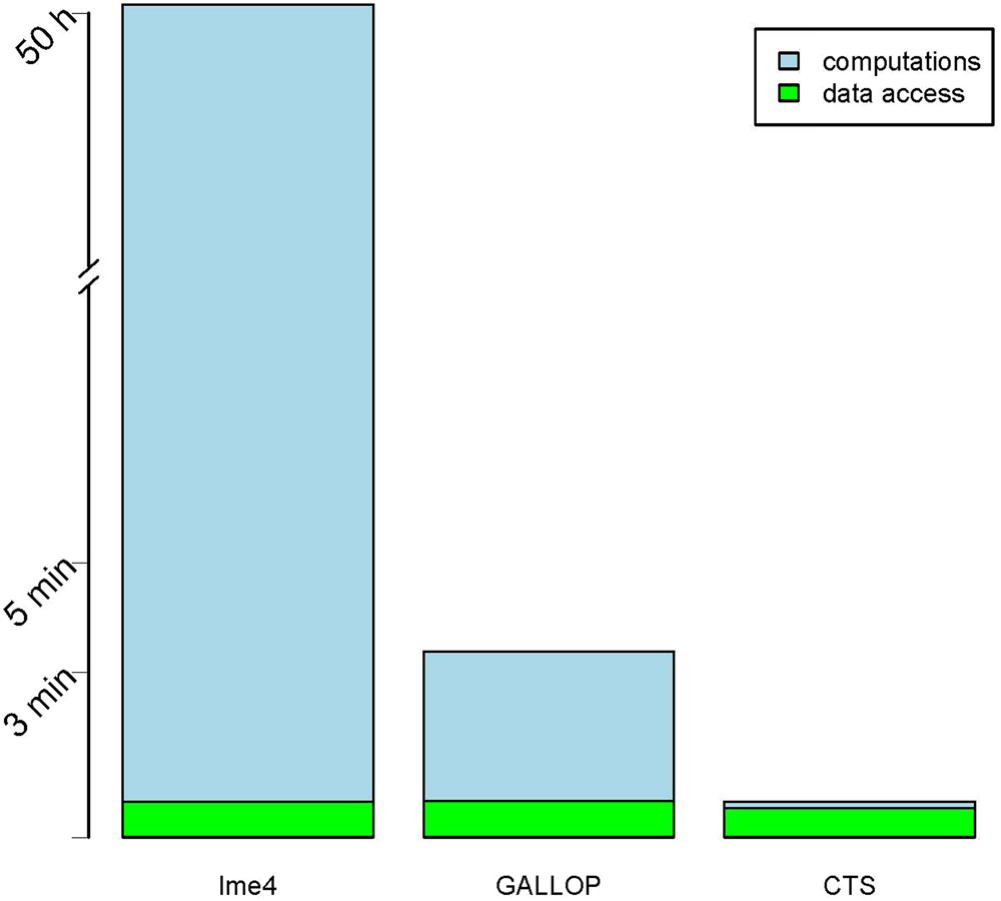
Time of the genome-wide analysis of the BMD data, 97384 SNPs from chromosome 22. Time spent on data access and time spent on computations are separated.

Our previous approach, conditional two-step (CTS) method combined with semi-parallel regression, computes p-values for the interaction effect about 15 times faster than the GALLOP. However, for CTS, SNP data access is still a bottleneck, 85% of the analysis time is spent on data access (Fig. 5). The genome-wide analysis of the BMD data was completed 5 times faster with CTS than with GALLOP. In case of very massive genome-wide analysis one could consider running CTS to filter out the least significant SNPs and proceed with GALLOP for more precise results.

GALLOP converts a genome-wide analysis with a longitudinal phenotype from a taxing multi-computer task to a job that can be run overnight on a single everyday computer. However, this is only true if access to the SNP data is fast enough. The memory limit in R depends on available RAM, but will usually not be larger than several gigabytes. The size of SNP data, even when split per chromosome, will exceed that size. GALLOP needs quick access to reasonably sized data blocks with multiple SNPs for all individuals. This is possible only when array-oriented binary files are used to store genotypes. We discussed this problem in detail, and proposed solutions in our previous work on fast analyses of cross-sectional outcomes^8^.

For correcting population stratification, in cross-sectional GWAS with possibly related individuals, mixed models are well established. Several algorithms have been proposed and implemented performing fast mixed model analysis in this framework. Multiple publications have proposed that this type of mixed models can be tweaked to analyze longitudinal data. Indeed, one may pretend that the repeated outcomes come from different pseudo-individuals and induce the correlation by passing the kinship matrix to the software. A quite extensive discussion on that topic is found in^10^. The author concludes that “the proper” longitudinal data analysis is to be preferred, but that it is too slow. Similarly, in ref.^11^. the authors analyzed longitudinal blood pressure data using EMMA, which tackles cross-sectional outcomes for related individuals. The authors tricked the software by mimicking an autoregressive structure in the kinship matrix. Although both papers study longitudinal data, their results touch only upon the main SNP effect. The interaction between SNP and time is not discussed.

Our algorithm assumes that the individuals are independent. An important extension is to adjust it for longitudinal data collected on related individuals, combining two types of mixed models. One approach to population stratification uses principal components of correlation matrix of the genotypes as covariates. They can be introduced as fixed effects in our model. The overhead is relatively small, because a large mixed model, without SNPs, is fitted once and each SNP is handled as a perturbation as described in the algorithm section.

The preferred approach would be to use multilevel modelling. Two sources of correlation then have to be combined: the temporal correlation between the repeated outcomes and the genetic correlation between the individuals. It would generate an additional random intercept, derived from the kinship matrix, which would destroy the sparseness of the estimating equations. But still the SNPs can be handled by perturbing a solution obtained without SNPs. This would be an interesting and fruitful topic for the future research.

## Methods

A linear mixed model for a longitudinal outcome which assumes random intercepts and slopes has the following hierarchical form^12^:

1 $$\{\begin{array}{l}{Y}_{i}={X}_{i}\beta +{Z}_{i}{b}_{i}+{\varepsilon }_{i},\,i=\mathrm{1,}\,\mathrm{...,}\,n\\ {b}_{i}\sim N\mathrm{(0,}\,D)\\ {\varepsilon }_{i}\sim N\mathrm{(0,}\,{{\rm{\Sigma }}}_{i})\\ {b}_{1},\,\mathrm{...,}\,{b}_{n},{\varepsilon }_{1},\,\mathrm{...,}\,{\varepsilon }_{n}\,{\rm{independent}}\mathrm{.}\end{array}$$

In (1) *Y*_*i*_ is *k*_*i*_ dimensional vector of responses for individual *i*, *X*_*i*_ is *k*_*i*_ × *p* matrix with all predictors, *Z*_*i*_ is *k*_*i*_ × 2 dimensional matrix with ones in the first column and *t*_*i*_ in the second column, *β* is a *p*-dimensional vector of coefficients identical for all individuals and *b*_*i*_ is a 2-dimensional vector containing the random effects. Measurement error is represented by the *k*_*i*_-dimensional vector *ε*_*i*_. Furthermore, *D* is the 2 × 2 variance-covariance matrix of random effects and Σ_*i*_ is *k*_*i*_ × *k*_*i*_ the variance-covariance matrix of measurement error. Typically, the unknown parameters, consisting of variances, fixed and random effects, are estimated using for example Newton-Raphson algorithm. However, if the variances are known, the fixed and random effects can be estimated simultaneously by solving a penalized least squares problem given by equations:

2 $$(\begin{array}{cc}X^{\prime} X & X^{\prime} Z\\ Z^{\prime} X & Z^{\prime} Z+P\end{array})(\begin{array}{c}\beta \\ b\end{array})=(\begin{array}{c}X^{\prime} y\\ Z^{\prime} y\end{array}).$$

In (2) matrices *X*, *Y*, and *b* are build of *X*_*i*_′s, *Y*_*i*_′s, and *b*_*i*_′s stacked underneath each other. Matrices *Z* and *P* are block diagonal with *Z*_*i*_ and *P*_*i*_ on the diagonals, where *P*_*i*_ = (*D*/*σ*^2^)^−1^. System (2) is similar to Henderson’s system of equations for mixed models.

A typical linear mixed model in a genome-wide association study will have a form:

3 $${Y}_{i}={\beta }_{0}+{\beta }_{1}{t}_{i}+{\beta }_{2}\,{{\rm{SNP}}}_{i}+{\beta }_{3}{t}_{i}\,{\rm{SNP}}{}_{i}+{C}_{i}{\beta }^{{\rm{COV}}}+{b}_{0i}+{b}_{1i}{t}_{i}+{\varepsilon }_{i},$$

where *t*_*i*_ is a *k*_*i*_-dimensional vector with measurement occasions, SNP_*i*_ is a *k*_*i*_-dimensional vector with SNP values (constant over time), and *C*_*i*_ is a *k*_*i*_ × *q*-dimensional matrix with constant or time-varying additional covariates (such as height, weight, age etc.). We call model (3) a full model. Additionally, the reduced model is constructed from (3) omitting the SNP effects, as given in (4)

4 $${Y}_{i}={\beta }_{0}^{\ast }+{\beta }_{1}^{\ast }{t}_{i}+{C}_{i}{\beta }^{{{\rm{COV}}}^{\ast }}+{b}_{0i}^{\ast }+{b}_{1i}^{\ast }{t}_{i}+{\varepsilon }_{i}^{\ast }.$$

The system of equations solving the penalized least squares problem for the reduced model have a special structure. We illustrate it for the case *n*= 3:5where:

$${S}_{i}=(\begin{array}{cc}\sum _{j}1 & \sum _{j}{t}_{ij}\\ \sum _{j}{t}_{ij} & \sum _{j}{t}_{ij}^{2}\end{array}),\,{b}_{i}^{\ast }=(\begin{array}{c}{b}_{0i}^{\ast }\\ {b}_{1i}^{\ast }\end{array})\,{\rm{and}}\,{r}_{i}=(\begin{array}{c}\sum _{j}{y}_{ij}\\ \sum _{j}{t}_{ij}{y}_{ij}\end{array})\mathrm{.}$$

Note that in (5) matrix $${X}^{\ast }=[\begin{array}{ccc}{\bf{1}} & {t}_{i} & {C}_{i}\end{array}]$$ and the * distinguishes which components of the model are altered (with respect to length and/or values) due to misspecified model (4). The above system has a block structure as divided by the solid lines and can be written as

6 $$(\begin{array}{cc}{A}_{11} & {A}_{21}^{^{\prime} }\\ {A}_{21} & {A}_{22}\end{array})(\begin{array}{c}{\beta }^{\ast }\\ {b}^{\ast }\end{array})=(\begin{array}{c}{q}_{1}\\ {q}_{2}\end{array}),$$

with the explicit solution given by:

7 $${\beta }^{\ast }={({A}_{11}-{A}_{21}^{^{\prime} }{A}_{22}^{-1}{A}_{21})}^{-1}({q}_{1}-{A}_{21}^{^{\prime} }{A}_{22}^{-1}{q}_{2})\,{\rm{and}}\,\,{b}^{\ast }={A}_{22}^{-1}({q}_{2}-{A}_{21}{\beta }^{\ast }\mathrm{).}$$

The P^*^ matrix can be easily obtained by fitting a mixed-effects model excluding SNP in any standard software (for example the R package **lme4**). The software does not explicitly return the P^*^, but it does return the variance-covariance matrix of the random effects (matrix *D*) and the variance of measurement error (*σ*^2^). In R matrix P^*^ is obtained by calling solve $(D/{\sigma }^{2})$.

An additional computational simplification can be obtained by ensuring that *A*_22_ in (7) is the identity matrix. This goal can be achieved as follows. Any system *Ab* = *q* can equivalently be solved by (*KAK*′)(*K*^−1^*b*) = *Kq*. Applied to (7), we get

8 $$(\begin{array}{cc}I & 0\\ 0 & {\rm{\Phi }}\end{array})(\begin{array}{cc}{A}_{11} & {A}_{21}^{^{\prime} }\\ {A}_{21} & {A}_{22}\end{array})(\begin{array}{cc}I & 0\\ 0 & {\rm{\Phi }}^{\prime} \end{array})(\begin{array}{cc}I & 0\\ 0 & {{\rm{\Phi }}}^{-1}\end{array})(\begin{array}{c}{\beta }^{\ast }\\ {b}^{\ast }\end{array})=(\begin{array}{cc}I & 0\\ 0 & {\rm{\Phi }}\end{array})(\begin{array}{c}{q}_{1}\\ {q}_{2}\end{array})$$

9 $$(\begin{array}{cc}{A}_{11} & ({\rm{\Phi }}{A}_{21})^{\prime} \\ {\rm{\Phi }}{A}_{21} & {\rm{\Phi }}{A}_{22}{\rm{\Phi }}^{\prime} \end{array})(\begin{array}{c}{\beta }^{\ast }\\ {{\rm{\Phi }}}^{-1}{b}^{\ast }\end{array})=(\begin{array}{c}{q}_{1}\\ {\rm{\Phi }}{q}_{2}\end{array})$$

thus, the goal is to choose Φ such that Φ*A*_22_Φ′ = *I*. Fortunately, *A*_22_ is block diagonal with each 2 × 2 block being equal to *S*_*i*_ + P^*^. Consequently, Φ is also block diagonal with 2 × 2 blocks Φ_*i*_. Then, we need to find Φ_*i*_ such that Φ_*i*_(*S*_*i*_ + P^*^)${{\rm{\Phi }}}_{i}^{^{\prime} }$ = *I*. Let *U*_*i*_Ω_*I*_${U}_{i}^{^{\prime} }$ be the eigendecomposition of *S*_*i*_ + P^*^, where *U*_*i*_ is the matrix of eigenvectors with ${U}_{i}^{^{\prime} }$*U*_*i*_ = *I* and Ω_*i*_ is the diagonal matrix of positive eigenvalues. Choose ${{\rm{\Phi }}}_{i}={{\rm{\Omega }}}_{i}^{-\mathrm{1/2}}{U}_{i}^{^{\prime} }$ and it is readily verified that

10 $${{\rm{\Phi }}}_{i}({S}_{i}+{P}^{\ast }){{\rm{\Phi }}}_{i}^{^{\prime} }={{\rm{\Omega }}}_{i}^{-\mathrm{1/2}}{U}_{i}^{^{\prime} }({S}_{i}+{P}^{\ast }){U}_{i}{{\rm{\Omega }}}_{i}^{-\mathrm{1/2}}={{\rm{\Omega }}}_{i}^{-\mathrm{1/2}}{U}_{i}^{^{\prime} }{U}_{i}{{\rm{\Omega }}}_{i}{U}_{i}^{^{\prime} }{U}_{i}{{\rm{\Omega }}}_{i}^{-\mathrm{1/2}}=I\mathrm{.}$$

The linearly transformed system becomes11

12 $$(\begin{array}{cc}{A}_{11} & {A}_{21}^{\mathrm{tra}{\rm{n}}^{\prime} }\\ {A}_{21}^{{\rm{tran}}} & I\end{array})(\begin{array}{c}{\beta }^{\ast }\\ \theta \end{array})=(\begin{array}{c}{q}_{1}\\ {q}_{2}^{{\rm{tran}}}\end{array})$$

with ${\theta }_{i}={{\rm{\Omega }}}_{i}^{\mathrm{1/2}}{U}_{i}^{^{\prime} }{b}_{i}$ and solutions

13 $${\beta }^{\ast }=({A}_{11}-{A}_{21}^{{\rm{tran}}^{\prime} }{A}_{21}^{{\rm{tran}}}{)}^{-1}({q}_{1}-{A}_{21}^{{\rm{tran}}^{\prime} }{q}_{2}^{{\rm{tran}}})\,\,{\rm{and}}\,\theta ={q}_{2}^{{\rm{tran}}}-{A}_{21}^{{\rm{tran}}}{\beta }^{\ast }\mathrm{.}$$

Note that the random effects have been transformed, such that ${b}_{i}^{\ast }={U}_{i}{{\rm{\Omega }}}_{i}^{-\mathrm{1/2}}{\theta }_{i}$. Usually, the solution for the subject-specific effects is not of interest in GWA analyses. Nevertheless, random intercepts and slope from the reduced model can be easily obtained from the lme4 package.

We add a SNP to the model, creating a border to the previous system of equations. Two effects, cross-sectional and longitudinal are added, so $$G=[\begin{array}{cc}{\rm{SNP}} & {\rm{SNP}}\ast t\end{array}]$$ is a Σ_*i*_*k*_*i*_ × 2 dimensional matrix, with SNP values repeated *k*_*i*_ times for all individuals in the first column and the SNP values repeated *k*_*i*_ times multiplied time occasions in the second column. Repeating SNP data for each individual *k*_*i*_ times seems like a time consuming step. However, SNP is constant over time and thus *G*_*i*_ = SNP_*i*_ * *Z*_*i*_. In our implementation the vector replicating SNP vector *k* times is never created explicitly. Regardless the value of *k*_*i*_ SNP values have to be replicated twice per individual resulting in additional efficiency. The augmented system of equation has the form:14where *β*^SNP^ = (*β*_2_, *β*_3_)′. Note that system (14) is just Henderson’s system for the full model, where *X* = [X^*^G] and the transformations have been used to simplify *Z*′*Z* + *P*. The transformation has been done based on P^*^ and not P, assuming that they are the same. This assumption does not strictly hold, but the approximation is very precise. In another article^13^ we showed that D^*^ of the SNP-model equals

15 $$(\begin{array}{cc}{\sigma }_{0}^{2}+{\beta }_{2}^{2}\,{\rm{var}}\,({\rm{SNP}}) & \rho {\sigma }_{1}{\sigma }_{2}+{\beta }_{2}{\beta }_{3}\,{\rm{var}}\,(\,{\rm{SNP}})\\ \rho {\sigma }_{1}{\sigma }_{2}+{\beta }_{2}{\beta }_{3}\,{\rm{var}}\,({\rm{SNP}}) & {\sigma }_{1}^{2}+{\beta }_{3}^{2}\,{\rm{var}}\,({\rm{SNP}})\end{array}).$$

When a SNP is not important in the model, i.e *β*_2_ and *β*_3_ are practically zero, D^*^ is essentially equal to *D*. This is the case for most of the SNPs in GWAS. In the situation when SNP has an effect (cross-sectional and/or longitudinal), the variances in D^*^ will be inflated. The cross-sectional effect inflates the variance of the random intercept, while the longitudinal effect affects the variance of the random slope. The magnitude of this inflation depends on the *β*_2_ and *β*_3_. The covariance in D^*^ is influenced only if both SNP effects are non-zero.

We can write the system (14) as

16 $$(\begin{array}{cc}{H}_{11} & {H}_{21}^{^{\prime} }\\ {H}_{21} & {H}_{22}\end{array})(\begin{array}{c}{\beta }^{{\rm{SNP}}}\\ \psi \end{array})=(\begin{array}{c}{J}_{11}\\ {J}_{21}\end{array}).$$

Solving system (16) for *β*^SNP^ gives us

17 $${\beta }^{{\rm{SNP}}}={({H}_{11}-{H}_{21}^{^{\prime} }{H}_{22}^{-1}{H}_{21})}^{-1}({J}_{11}-{H}_{21}^{^{\prime} }{H}_{22}^{-1}{J}_{21}\mathrm{).}$$

It may seem that, in (17), ${H}_{22}^{-1}{J}_{21}$ and ${H}_{22}^{-1}{H}_{21}$ are expensive operations, since they involve inverting (2*n* + *p* + 2) × (2*n* + *p* + 2) dimensional matrix. However, the inverse of *H*_22_ is not needed explicitly. Note that ${H}_{22}^{-1}{J}_{21}$ is a matrix with two columns, containing solutions of system (13). It can be computed once and stored. The second operation is a solution for the mixed model given in (13) but for a different right hand side, namely *H*_21_. Note that in this case the RHS of the system is two-dimensional.

### Standard errors

To compute the variance-covariance matrix of the estimated fixed and random effects in a mixed model we need to invert the LHS matrix of system (2). Standard errors are equal to the square roots of the diagonal elements of that matrix. In penalized least squares notation, we need to invert LHS of system (2) and multiply diagonal elements by *σ*^2^. In our case we are interested only in the inference for SNP effects. They are the upper-left part of the expression

18 $${\hat{\sigma }}^{2}{(\begin{array}{cc}{H}_{11} & {H}_{21}^{^{\prime} }\\ {H}_{21} & {H}_{22}\end{array})}^{-1}\mathrm{.}$$

Using the formula for the matrix inverse in block form, the standard errors of *β*^SNP^ are given by

19 $$\hat{\sigma }\sqrt{(\,{\rm{diag}}\,{({H}_{11}-{H}_{21}^{^{\prime} }{H}_{22}^{-1}{H}_{21})}^{-1})}\mathrm{.}$$

Note that this diagonal has already been computed in (17) showing that the computation of the standard errors is trivial.

### Missing phenotype

Mixed models handle unbalanced data with ease; all subjects, whatever their number of observations, are taken into the analysis. In this sense the concept of missing data in case of mixed models does not exist. However, our algorithm assumes that the phenotype data for every subject consists of *k* rows and that some of the values are missing (coded as NA). To properly estimate the solution of a mixed model the weighting matrix has to be introduced

20 $$(\begin{array}{cc}X^{\prime} WX & X^{\prime} WZ\\ Z^{\prime} WX & Z^{\prime} WZ+P\end{array})(\begin{array}{c}\beta \\ b\end{array})=(\begin{array}{c}X^{\prime} Wy\\ Z^{\prime} Wy\end{array})\mathrm{.}$$

Matrix W is a diagonal *nk* × *nk* matrix with 0 or 1 in the diagonal indicating if the observation is valid or not. Note that in practice matrix W does not have to be build, since applying weights is equivalent to replacing rows with missing data by all zeros.

### Implementation

GALLOP is implemented in one relatively short R program, provided in the Supplementary Materials. An important computing challenge was to avoid repeating each SNP value *k* times, to be able to calculate cross products in the border matrices. We achieved this by storing the basis of those matrices, calculated using Kronecker products, in a vector instead of a matrix. This way we can summarize the SNP state directly with two numbers per individual, regardless of *k*.

### Data availability

The BMD data are a part of Rotterdam Study and are confidential. The scripts used to generate the data in the simulation study are available from the corresponding author upon request.

## Electronic supplementary material

Supplementary Material
