# Supplementary material for: Genome-wide Analysis of Large-scale Longitudinal Outcomes using Penalization —GALLOP algorithm
Source: Sci Rep. 2018 May 1;8:6815. doi: 10.1038/s41598-018-24578-7 (PMC5931565; doi:10.1038/s41598-018-24578-7)
Supplement: Supplementary file 1 — Supplementary Material [file 41598_2018_24578_MOESM1_ESM.docx]

**Supplementary Material**

*Genome-wide Analysis of Large-scale Longitudinal Outcomes using Penalization – GALLOP algorithm*

Karolina Sikorska, Emmanuel Lesaffre, Patrick J.F. Groenen, Fernando Rivadeneira,
 and Paul H.C. Eilers

**This file provides R implementation of GALLOP algorithm.**

### To deal with potential multicolinearity caused by adding interaction effect of SNP x time, global centering of time variable can be performed as a preparation.

Data$time <- Data$time – mean(Data$time)

*# Fit model without SNP using lmer, covs are names of all additional covariates in the model*

mod_formula <- as.formula(paste("y ~ time +", paste(covs, collapse= "+"), "+ (time|id)"))

mod1 = lmer(mod_formula, data = Data, na.action = "na.omit")

*# Extract variance components and compute penalty matrix (P)*

varcor = VarCorr(mod1)

G = varcor$id

sig = attr(varcor, "sc")

P = solve(G / sig ^ 2)

# Put data in convenient arrays and vector

TT = XY[ , 1 : 2]

nxy = ncol(XY)

X = XY[ , -nxy]

nx = ncol(X)

y = XY[ , nxy]

*# Compute components of block-diagonal system with covariates (without SNP)*

*# Additionally compute and store object SS = Rot %*% Si, which is used later on*

A21 = matrix(NA, 2 * n , 2 + ncov)

q2 = rep(NA, 2 * n)

SS = matrix(NA, 2 * n , 2)

for (i in 1 : n ) {

uk = (i - 1) * k + (1 : k)

u2 = (i - 1) * 2 + (1 : 2)

Ti = TT[uk, ]

Si = crossprod(Ti, Ti)

sv = svd(Si + P)

Rot = sqrt(1 /sv$d) * sv$u

Q = Rot %*% t(Ti)

SS[u2, ] = Rot %*% Si

A21[u2, ] = Q %*% X[uk, ]

q2[u2] = Q %*% y[uk]

}

q1 = crossprod(X, y)

A11 = crossprod(X)

# Solve the system (20)

Q = A11 - crossprod(A21)

q = q1 - crossprod(A21, q2)

sol = solve(Q, q)

blups = q2 - A21 %*% sol

*# Compute sums of products per subject involved in the crossprod(X, G), crossprod(G) and crossprod(G, y). We use row-wise Kronecker product to avoid repeating SNP vector k times*

ex = matrix(1, 1, ncov + 2)

et = matrix(1, 1, 2)

XTk = kronecker(et, X) * kronecker(TT, ex)

TTk = kronecker(et, TT) * kronecker(TT, et)

Tyk = y * TT

XTs = matrix(0, n, ncol(XTk))

TTs = matrix(0, n, ncol(TTk))

Tys = matrix(0, n, 2)

AtS = matrix(0, n, 2 * nx)

for (i in 1:n) {

uk = (i - 1) * k + (1 : k)

XTs[i, ] = apply(XTk[uk, ], 2, sum)

TTs[i, ] = apply(TTk[uk, ], 2, sum)

Tys[i, ] = apply(Tyk[uk, ], 2, sum)

u2 = (i - 1) * 2 + (1 : 2)

AtS[i, ] = c(crossprod(A21[u2, ], SS[u2, ]))

}

*# Add SNPs one by one and solve*

Theta = D = matrix(NA, ns, 2)

for (i in 1 : ns) {

si = SNPS[, i]

snp2 = rep(si, each = 2)

H1 = matrix(crossprod(si, XTs), nx, 2)

H2 = snp2 * SS

AtH = matrix(crossprod(si, AtS), nx, 2)

R = H1 - AtH

Cfix = solve(Q, R)

Cran = H2 - A21 %*% Cfix

GtG = matrix(crossprod(si ^ 2, TTs), 2, 2)

Gty = matrix(crossprod(si, Tys), 2, 1)

V = GtG - crossprod(H1, Cfix) - crossprod(H2, Cran)

v = Gty - crossprod(H1, sol) - crossprod(H2, blups)

Theta[i , ] = solve(V, v)

D[i, ] = diag(solve(V))

}

SE = sqrt(sig2) * sqrt(D)

Pval = 2 * pnorm(-abs(Theta / SE))
